# Effectiveness and Safety of Recombinant Type III Humanized Collagen Solution Injection Combined With Collagen‐III Multi‐Peptide Serum in Improving Signs of Photoaging: A Prospective, Split‐Face Controlled Clinical Trial

**DOI:** 10.1111/jocd.70857

**Published:** 2026-05-05

**Authors:** Xiaolei Qin, Jinlong Zhai, Lingyan Liu, Hong Shu, Lin Zhu, Qi Shi, Yining Luo

**Affiliations:** ^1^ Department of Dermatology DeYi Skin DeYue Clinic Shenzhen China

## Abstract

**Background:**

Skin aging is a multifactorial process involving the progressive deterioration of skin structure and function, marked by collagen degeneration, impairing the structural integrity of the dermis. Intradermal injection of recombinant type III humanized collagen (RhCol‐III) solution is a promising anti‐aging strategy through direct collagen replenishment, but its synergistic effects when combined with a collagen‐III multi‐peptide serum remain unexplored.

**Aims:**

This study evaluates the effectiveness and safety of a RhCol‐III solution injection combined with a collagen‐III multi‐peptide serum in improving photoaging.

**Methods:**

A prospective, single‐center, randomized, split‐face, single‐blind trial was conducted. Participants received an intradermal injection of 2 mg/mL RhCol‐III solution with application of collagen‐III multi‐peptide serum on the test side and intradermal injection of RhCol‐III solution only on the control side. The 12‐week follow‐up period assessed effectiveness and safety, with the primary endpoint being the participant‐reported skin satisfaction score (FACE‐Q satisfaction with skin) at 4 weeks post‐treatment.

**Results:**

A total of 54 participants were enrolled. The primary endpoint, FACE‐Q skin satisfaction score at week 4, significantly improved on both tested and control sides by 40.0% vs. 34.8% from baseline (both *p* < 0.0001; *p* < 0.05 between sides). By week 12, the tested side showed significantly better outcomes versus the control side in FACE‐Q skin satisfaction scores, GAIS significant improvement rates, improvement rates of fine lines and skin firmness (all *p* < 0.05). No adverse events related to the RhCol‐III solution injection were observed.

**Conclusions:**

The combination of injectable RhCol‐III and topical collagen‐III multi‐peptide serum is effective and safe in ameliorating photoaging of the forehead, periorbital and cheek areas among the Chinese population.

## 1 Introduction

Skin aging is a multifactorial process characterized by visible changes such as wrinkles and thinning [1]. This process manifests through various mechanisms, including the loss of facial soft tissue volume, gravitational ptosis of soft tissues, repetitive muscle movements, and degeneration of bone tissue [2]. Photoaging, induced by ultraviolet (UV) radiation, along with intrinsic aging, contributes to the reduction of collagen fibers, alteration of structural integrity, and diminishment of elasticity and volume in the skin. Specifically, these features present as roughened skin texture, deepened and widened wrinkles, increased skin laxity, and enlarged pores [1, 3].

To address these concerns, chemical peeling, energy‐based devices (including lasers, radiofrequency, and intense pulsed light), and intradermal injections are commonly used. For chemical peeling, superficial agents like glycolic acid and salicylic acid are frequently used; however, they may cause erythema, swelling, stinging, and potential post‐treatment effects including crusting and pigmentation changes [4]. Energy‐based devices, encompassing lasers, radiofrequency, and intense pulsed light, offer varying efficacies and respective risks [5]. While ablative lasers provide significant effectiveness, they pose risks such as infection and scarring. Advances in non‐ablative laser and radiofrequency technologies have enhanced treatment efficacy and safety [6]. These methods generally exhibit slower immediate effectiveness and carry a risk of adverse reactions [5, 7]. Thus, treatments that can deliver targeted hydration and nourishment directly to the dermis, promoting skin rejuvenation while minimizing adverse effects, are needed to address photoaging effectively.

Intradermal micropuncture injection is a minimally invasive technique that involves the injection of hydrating or nutritive substances into the dermis or subcutaneous connective tissues [8]. Hyaluronic acid‐based products have been extensively used in such injections, also known as “skin boosters,” due to their ability to boost structural integrity of the dermis and improve the biomechanical functions by retaining moisture, enhancing hydration, and improving skin elasticity [8]. Besides hyaluronic acid‐based products, intradermal micropuncture injection of collagen‐based products has also recently emerged in anti‐aging practices [9, 10].

Collagens serve as fundamental structural components of the extracellular matrix (ECM) in human skin, playing crucial roles in mechanical support, cell adhesion, cell differentiation, cell migration, ECM synthesis, as well as reducing inflammatory responses and inhibiting melanogenesis [1, 8, 11]. With advancing age and exposure to UV, endogenous collagen levels diminish, leading to a loss of skin elasticity and firmness [3]. Currently, the collagen‐based products used in the field of aesthetics include animal‐derived collagen and recombinant human collagen. The latter's amino acid sequence closely mirrors that of native human collagen, allowing seamless integration into pre‐existing biochemical processes such as extracellular matrix construction and repair. This similarity also reduces the risk of immune reactions, thereby enhancing product safety [12].

Collagen‐based products also encompass those containing type I collagen and type III collagen [13]. Type I collagen provides strong support to the skin, commonly used for deep filling and shaping; type III collagen has good elasticity and softness, which is more conducive to skin repair and regeneration, and is often used for superficial filling and improving skin texture [13]. Although there have been some case series published on the effects of RhCol‐III in skin repair and rejuvenation, there is a lack of full‐scale and controlled clinical studies to assess the long‐term effects and safety of the injectable material [14, 15, 16].

Integrated skincare refers to the use of scientifically validated effective skincare products before, during, and/or after aesthetic procedure treatment, which can improve the initial skin condition [11]. The goal is to enhance treatment effectiveness, minimize side effects, prolong results, and boost patient satisfaction [11]. A potential example of this integrated skincare regimen is the combination of RhCol‐III solution injection with a collagen‐III multi‐peptide serum. The injection delivers RhCol‐III directly into the dermis, providing cellular microenvironment support and stimulating intrinsic collagen production. Simultaneously, the daily use of a topical collagen‐III multi‐peptide serum is designed to nourish the epidermal layer, promote sustained collagen synthesis over time, and reinforce the skin barrier function. We believe that the dual approach may leverage the synergistic effects between deep tissue rejuvenation and superficial skin conditioning and consequently amplify the outcomes achievable by either intervention alone [6, 8, 11].

This study aims to evaluate the effectiveness and safety of a RhCol‐III solution injection used in conjunction with collagen‐III multi‐peptide serum. It seeks to explore the synergistic anti‐photoaging effects of injectables and integrated skincare approaches within the Chinese female population.

## 2 Materials and Methods

### 2.1 Study Design and Population

This study was a prospective, single‐center, randomized, split‐face, single‐blind, clinical trial conducted from March 2024 to August 2024. Signed informed consent and agreement to follow‐up visits were obtained before the study initiation. The study was approved by a Clinic Ethics Committee. Additionally, the study is part of an online clinical trial database.

The trial enrolled female participants who met the following inclusion criteria: (1) age 30 to 55 years; (2) had rough and dry facial skin with noticeable fine lines, and (3) Glogau photoaging graded II to IV, as assessed by investigators.

Exclusion criteria included: (1) facial scars, active dermatological conditions, unhealed wounds, or progressive skin diseases; (2) plans to undergo/current facial surgeries/treatments that could affect trial outcomes; (3) severe/genetic allergies, desensitization therapy plans during the study, or allergies to components of the investigational products; (4) coagulation disorders, use/planned use of anticoagulants, antiplatelet agents, or thrombolytics within 14 days prior to screening; (5) pregnancy/breastfeeding, or planning to become pregnant during the trial; (6) significant organ disease or active autoimmune diseases; (7) hypertrophic/keloid‐prone scars; (8) participation in other clinical trials within 30 days prior to screening; (9) any other conditions deemed unsuitable for participation by the investigator. Only participants meeting all inclusion criteria and none of these exclusion criteria were eligible for enrollment in this study.

### 2.2 Intervention

Following screening and enrollment, participants underwent a single session of recombinant type III humanized collagen solution injection (RhCol‐III; 2.0 mg/mL, 2.0 mL/vial; NMPA medical device registration number: 20233131245) with a total dose of 12 mL across the forehead (2–3 mL), periorbital area (including the infraorbital and eyelid regions, 3‐4 mL) and cheeks (6 mL). On the tested side, after injection with RhCol‐III solution, participants applied twice daily topical collagen‐III multi‐peptide serum (Collagen‐III Amplifier, Skinceuticals Inc.). On the control side, participants were only treated with the RhCol‐III solution injection, and did not receive the topical collagen serum. As part of their regular skincare routine, study participants applied a standard moisturizer twice a day and a standard sunscreen in the daytime on both sides of the face.

### 2.3 Effectiveness and Safety Evaluation

Follow‐up visits were scheduled for 12 weeks post‐injection, and assessments were conducted at four time points post‐injection: 2 weeks ±3 days, 4 weeks ±5 days, 8 weeks ±2 weeks, and 12 weeks ±2 weeks. The primary effectiveness endpoint of this trial was the participant skin satisfaction score (FACE‐Q Satisfaction with Skin) at 4 weeks post‐treatment. Secondary effectiveness endpoints included: participant skin satisfaction scores at 2, 8, and 12 weeks post‐injection; the significant improvement rate in Global Aesthetic Improvement Scale (GAIS) scores assessed by blinded independent investigators and participants at 2, 4, 8, and 12 weeks post‐treatment, where significant improvement was defined as a GAIS score of 1 to 2, with the significant improvement rate calculated as the number of participants showing significant improvement divided by the total number of participants, multiplied by 100%; the fine line improvement rate evaluated by blinded independent investigators using the Skin Aging Atlas at 2, 4, 8, and 12 weeks post‐treatment, with improvement defined as an increase of at least one grade on the Atlas scale, and the improvement rate calculated as the number of improved cases divided by the total number of participants, multiplied by 100%; skin elasticity and skin firmness compared to baseline, as measured by Multi‐Probe Adapter System (MPA) with the Cutometer (Courage + Khazaka Electronic GmbH, Cologne, Germany), using the MPA580 probe at 2, 4, 8, and 12 weeks post‐treatment. Safety assessments were primarily based on the incidence of adverse events (AEs) and treatment‐related adverse events.

### 2.4 Statistics

Statistical analyses were conducted using SAS EG 8.2 or higher. Quantitative data were summarized as mean ± standard deviation (SD) when approximately normally distributed and as median (first quartile [Q1], third quartile [Q3]) when non‐normally distributed. Categorical variables were presented as frequencies and percentages. Normality was assessed using the Shapiro–Wilk test.

For paired comparisons, including (1) comparisons between the tested and control sides within the same participant and (2) within‐side comparisons across time points, the paired *t*‐test or Wilcoxon signed‐rank test was used as appropriate based on data distribution. A two‐sided *p*‐value < 0.05 was considered statistically significant.

All randomized participants who received treatment were included in the Full Analysis Set (FAS), and all participants who received treatment and underwent safety evaluations were included in the Safety Set (SS).

## 3 Results

### 3.1 Patient Characteristics and Baseline Demographics

A total of 54 participants were enrolled. Ultimately, 52 participants completed the study. One participant withdrew due to unwillingness to continue with the clinical trial, while another was withdrawn by the investigator following AEs (acneiform rash and skin irritation), deeming it unsuitable for continued participation.

Based on the FAS, the baseline demographic characteristics of the participants are as follows: the mean age was 35.3 ± 5.72 years, ranging from 30 to 53 years, and most of the studied population was Han Chinese (92.6%, *n* = 50), while 7.4% (*n* = 4) belonged to other ethnicities. Regarding Glogau photoaging classification, 61.1% (*n* = 33) of participants were classified as type II, 37.0% (*n* = 20) as type III, and 1.9% (*n* = 1) as type IV; no participants were classified as type I (Table 1).

**TABLE 1:** Demographic and baseline characteristics of study participants.

	Total (*N* = 54)
Age (years)*	35.3 ± 5.72
Ethnicity, *n* (%)	
Chinese	54 (100)
Height (cm)*	161.0 ± 4.36
BMI (kg/m^2^)*	21.33 ± 2.91
Glogau photoaging classification	
Type I, *n* (%)	0
Type II, *n* (%)	33 (61.1)
Type III, *n* (%)	20 (37.0)
Type IV, *n* (%)	1 (1.9)

*Note:* *Plus‐minus values represent mean ± SD.

### 3.2 Effect of RhCol‐III on Participant Skin Satisfaction Scores (FACE‐Q and GAIS)

The primary effectiveness endpoint, participant skin satisfaction score (FACE‐Q Satisfaction with Skin) at Week 4, was significantly improved in both the tested and control sides. The median satisfaction scores were 63 (55, 76) for the tested side and 63 (51, 72) for the control side, representing improvements of 40.0% and 34.8% from baseline, respectively (both *p* < 0.0001) (Table 2). These results demonstrate that the RhCol‐III solution injection, used in conjunction with collagen‐III multi‐peptide serum, significantly enhances participant skin satisfaction. The distribution of scores showed a statistically significant difference, indicating a shift toward more positive values on the tested side (*p* < 0.05).

**TABLE 2:** Participants' FACE‐Q skin satisfaction score—primary effectiveness endpoint.

	Tested side (TS, *N* = 54)	Control side (CS, *N* = 54)	*p*
Baseline	43.0 (36.0, 49.0)^◊^	43.0 (36.0, 49.0)^◊^	0.4520^c^
Week 4	63.0 (55.0, 76.0)^△^	63.0 (51.0, 72.0)^△^	
TS vs. CS	0.0 (95% CI: 0.0, 2.0)^a^		0.0008^c^
Improvement rate^b^ after 4‐week treatment (%)	40.0 (15.1, 104.7)	34.8 (15.1, 84.6)	0.0499^c^

*Note:* TS ‐ test side, CS ‐ control side. ♢ Values represent median (Q1,Q3). △ Compared to baseline *p* < 0.0001. a. Value represents median difference. b. Improvement rate = (follow‐up results ‐ baseline results)/baseline results * 100%. c. Calculated using the Wilcoxon signed‐rank test.

**FIGURE 1:**
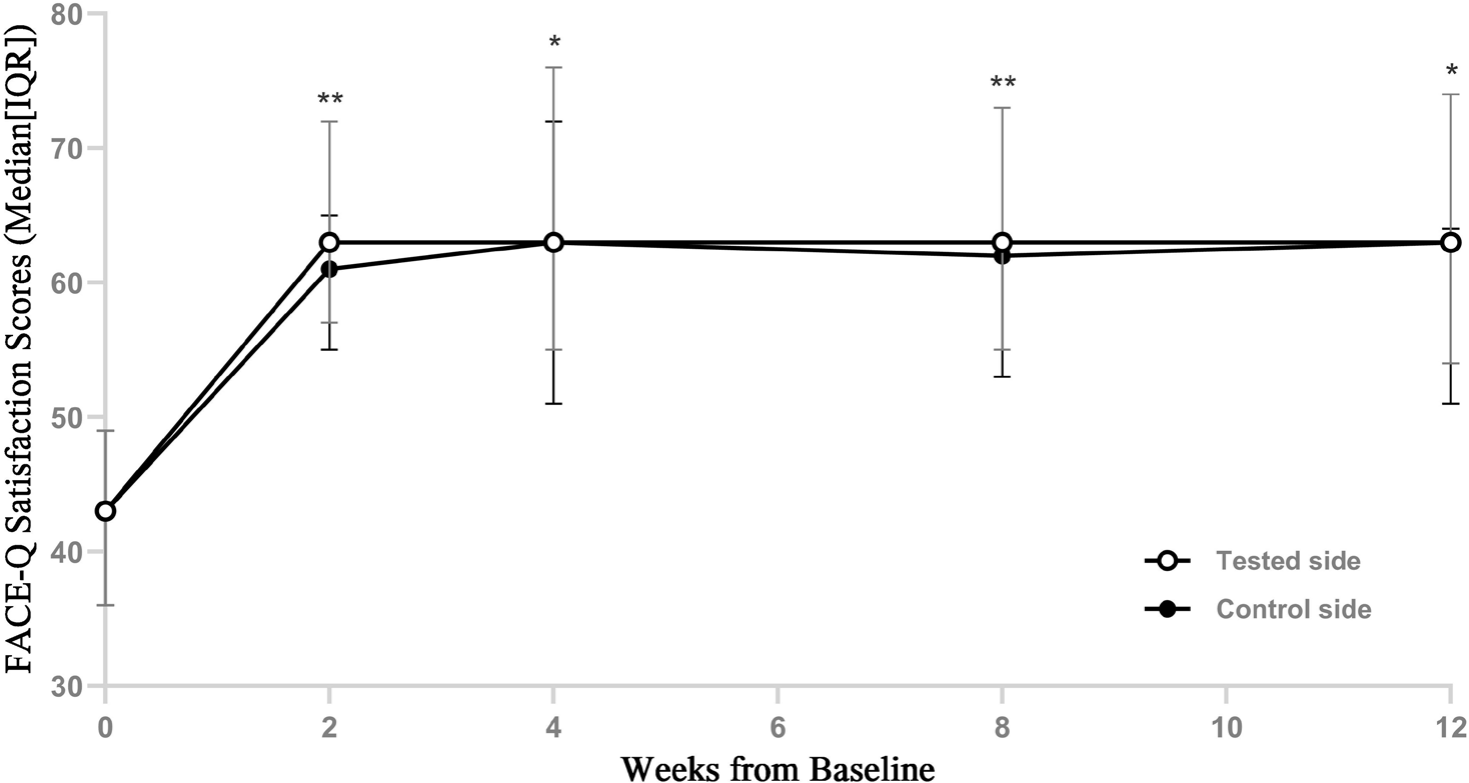
Participants' FACE‐Q satisfaction scores at each follow‐up visit. *p < 0.05; **p < 0.001.

At 2‐, 8‐, and 12‐weeks post‐treatment, FACE‐Q satisfaction scores showed significant improvements from baseline (all *p* < 0.0001). Comparisons of FACE‐Q satisfaction scores between the two sides revealed that scores on the tested side were consistently higher than those on the control side at each follow‐up visit, with statistically significant differences (*p* < 0.05) (Figure 1).

**FIGURE 2:**
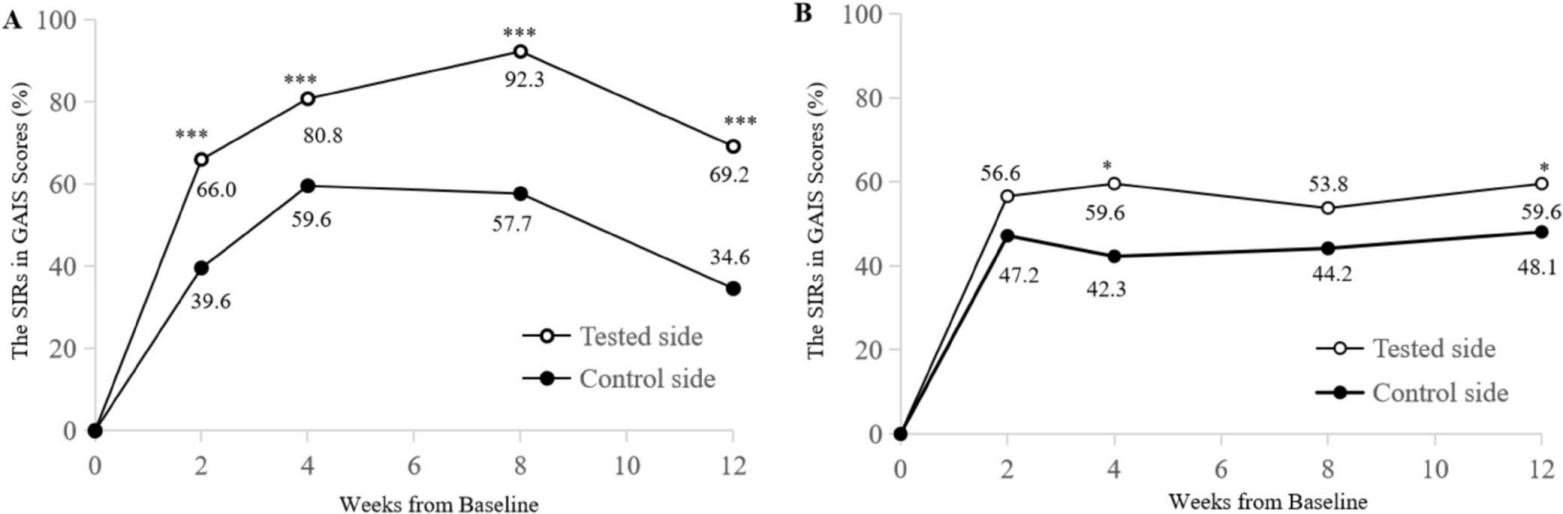
Significant Improvement Rates (SIRs, %) in Global Aesthetic Improvement Scale (GAIS) scores over time. (A) The SIRs in GAIS scores assessed by blinded independent investigators. (B) The SIRs in GAIS scores self‐assessed by participants. The criterion for ‘significant improvement’ was a GAIS score of 1–2 points. *p < 0.05; ***p < 0.0001.

At 2‐, 4‐, 8‐, and 12‐weeks post‐treatment, the significant improvement rates in GAIS scores assessed by blinded independent investigators for the tested side were 66.0% (35/53), 80.8% (42/52), 92.3% (48/52), and 69.2% (36/52), respectively. For the control side, the rates at the same timepoints were 39.6% (21/53), 59.6% (31/52), 57.7% (30/52), and 34.6% (18/52), respectively (Figure 2A). Comparisons of GAIS significant improvement rates between the two sides showed that the tested side consistently exceeded the control side at all follow‐up visits, with statistically significant differences observed (*p* < 0.05).

At 2‐, 4‐, 8‐, and 12‐weeks post‐treatment, the significant improvement rates in GAIS scores self‐assessed by participants for the tested side were 56.6% (30/53), 59.6% (31/52), 53.8% (28/52), and 59.6% (31/52), respectively. For the control side, the rates at the same timepoints were 47.2% (25/53), 42.3% (22/52), 44.2% (23/52), and 48.1% (25/52), respectively (Figure 2B). Comparisons of GAIS significant improvement rates between the two sides proved a numerical difference between the two sides at 2‐ and 8‐weeks post‐treatment; however, at 4‐ and 12‐weeks post‐treatment, a difference was both numerical and also statistically significant (*p* < 0.05).

### 3.3 Effect of RhCol‐III on Facial Fine Lines and Wrinkles

**FIGURE 3:**
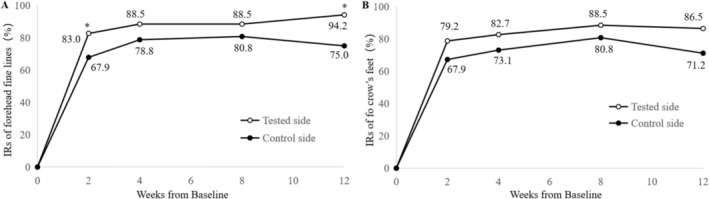
Improvement rates (IRs) of forehead fine lines and crow's feet over time. (A) IRs of forehead fine lines. (B) IRs of crow's feet. Improvement was defined as an increase of ≥ 1 point in Skin Aging Atlas scale compared to baseline; IR = the number of improved cases/the number of participants × 100%. *p < 0.05.

**FIGURE 4:**
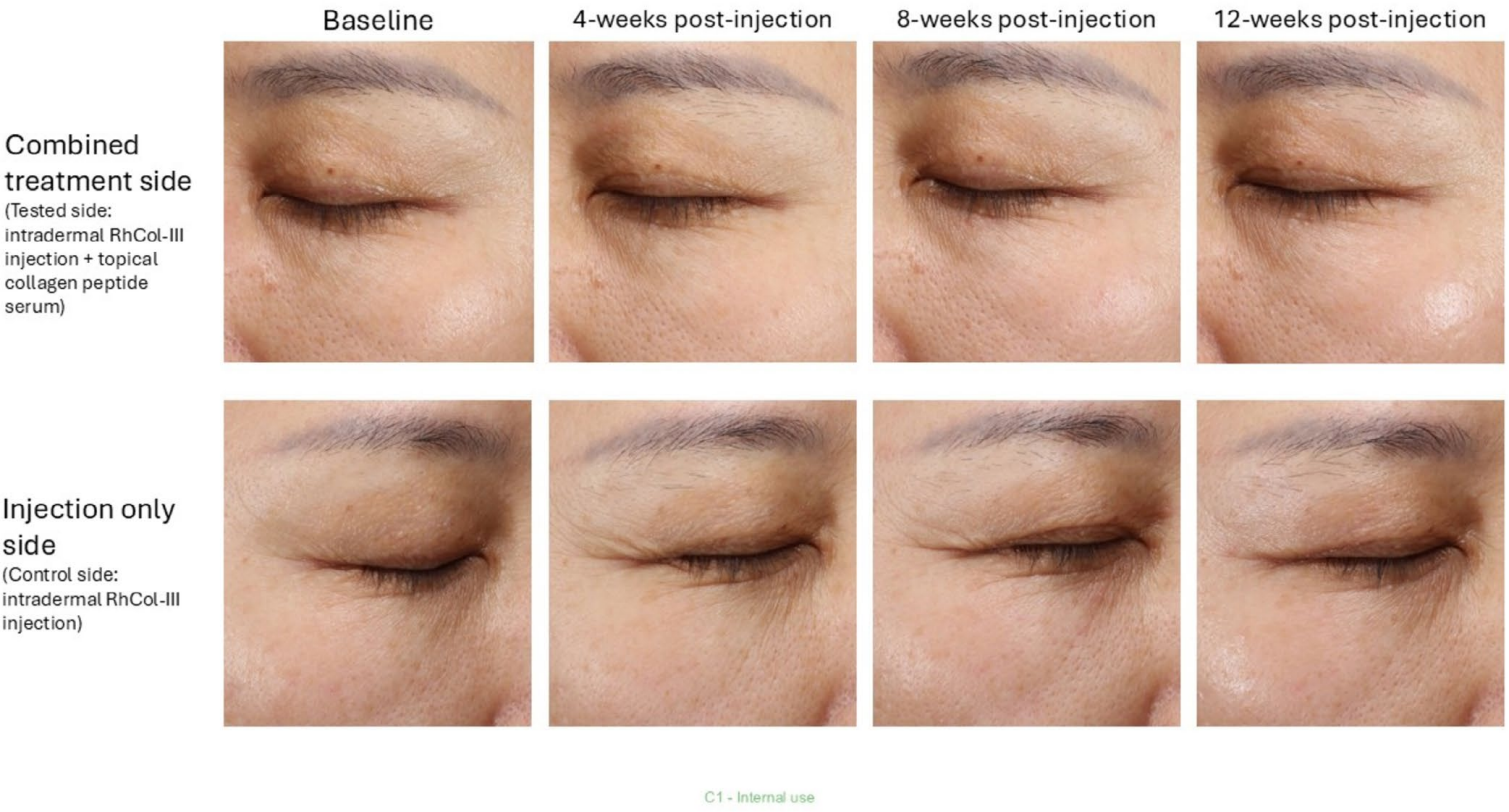
Significant qualitative improvement of fine lines and crow's feet on both sides of the face in a study participant over time. Tested side, or combination therapy side (RhCol‐III injection with topical collagen serum application) showcased a more significant aesthetic improvement, as compared to the control side, or monotherapy side (RhCol‐III injection alone).

At 2, 4, 8, and 12 weeks post‐treatment, the improvement rates of forehead fine lines on the tested side were 83.0% (43/53), 88.5% (46/52), 88.5% (46/52), and 94.2% (49/52), respectively, compared with 67.9% (36/53), 78.8% (41/54), 80.8% (42/54), and 75.0% (39/54) on the control side (Figure 3A). At 2‐ and 12‐weeks post‐treatment, the improvement rates on the tested side for forehead fine lines were significantly higher than those on the control side (*p* < 0.05). For crow's feet, the improvement rates on the tested side at 2, 4, 8, and 12 weeks post‐treatment were 79.2% (42/53), 82.7% (43/52), 88.5% (46/52), and 86.5% (45/52), respectively, compared to 67.9% (36/53), 73.1% (38/52), 80.8% (42/52), and 71.2% (37/52) on the control side (Figure 3B). Despite the fact that there was no statistically significant difference between the two sides, the improvement in crow's feet was still more pronounced on the test side than on the control side (*p* > 0.05). As shown in Figure 4, at 4‐, 8‐, and 12‐weeks post‐treatment participants exhibited notable improvements in the periorbital fine lines and crow's feet on the test side, with reductions in both the length and depth.

### 3.4 Effect of RhCol‐III on Facial Skin Firmness and Elasticity

**FIGURE 5:**
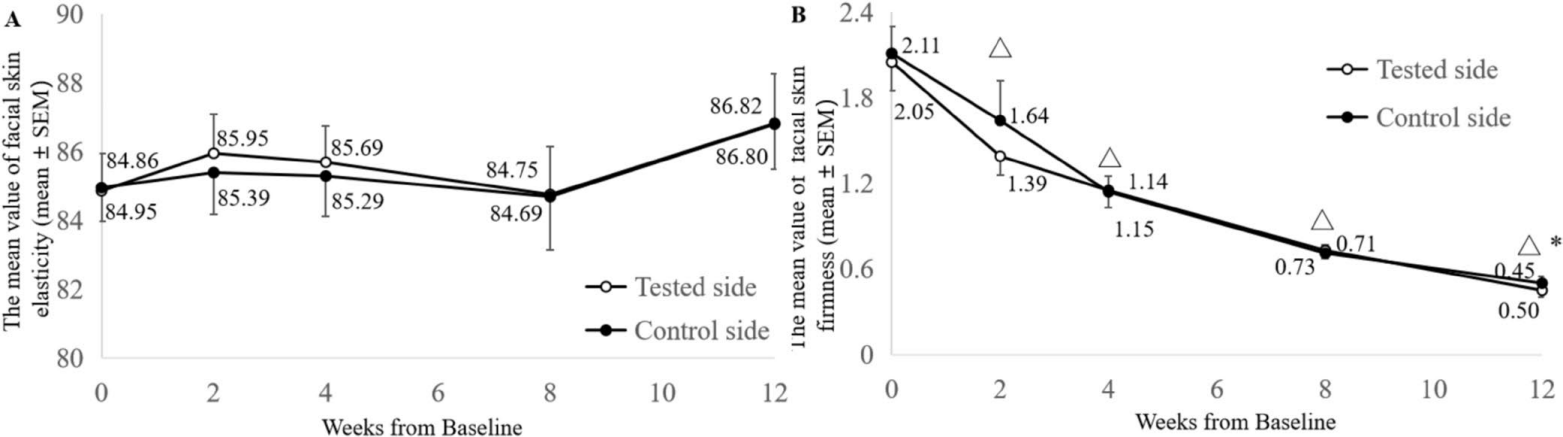
Mean values of skin elasticity (A) and skin firmness (B) over time. *Between‐group comparison, *p* < 0.05. △ Compared with baseline, *p* < 0.0001.

For skin firmness, at baseline and at 2, 4, 8, and 12 weeks post‐treatment, the mean values on the tested side were 2.05 ± 1.43, 1.39 ± 0.93, 1.15 ± 0.83, 0.73 ± 0.44, and 0.45 ± 0.36, respectively, compared to 2.11 ± 1.43, 1.64 ± 2.04, 1.14 ± 0.80, 0.71 ± 0.43, and 0.50 ± 0.38 on the control side (Figure 5B). The skin firmness value reflects the area under the envelope curve formed during the application and release of negative pressure throughout the testing period. A smaller value indicates tighter skin, so improvements in skin firmness are represented by a negative change rate. Both sides demonstrated statistically significant improvements in skin firmness from baseline at all follow‐up time points (*p* < 0.05). Furthermore, there was a statistically significant difference in skin firmness between the two sides at 12 weeks (*p* < 0.05).

With regards to improvements in skin elasticity, both sides of the face showed a positive trend at week 12, as compared to baseline (*p* > 0.05, Figure 5A).

### 3.5 Safety

A total of 54 participants were enrolled in this trial, all of whom were included in the SS. Based on the SS, no RhCol‐III injection‐related adverse events were reported. Participants were specifically monitored for common injection‐site reactions, including erythema, swelling, pain, bruising, nodules, and hypersensitivity, with an incidence of 0.0% for all categories. One participant (1.9%) reported a mild injection‐related adverse reaction unrelated to the product material itself, which resolved after facial mask application. Regarding the collagen peptide serum: one participant (1.9%) experienced serum‐related adverse events, specifically mild acneiform rash and skin irritation (manifesting as dryness and stinging). These symptoms occurred at week 2 and led to the discontinuation of the serum. The symptoms resolved completely following treatment with topical desonide cream. No serious adverse events (SAEs) related to either test product were observed.

## 4 Discussion

To our knowledge this is the first prospective, split‐face clinical trial to evaluate the effectiveness and safety of combining RhCol‐III solution injection with topical collagen‐III multi‐peptide serum in improving skin quality in Chinese female participants. The results demonstrate that, during the 12 weeks post‐treatment follow‐up period, significant improvements from baseline were consistently observed following injections of the RhCol‐III solution on both sides of the face, with the tested side demonstrating an added‐on benefit of the topical collagen serum. The primary endpoint, FACE‐Q skin satisfaction score at Week 4, significantly improved on both tested and control sides, with a statistically significant difference favoring the tested side over the control side. By Week 12 post‐treatment, the test side showed comparatively better improvement in skin surface evenness, as represented by more significant improvement in fine lines and skin firmness. These results demonstrate that combining collagen injection and topical collagen effectively ameliorate the signs of photoaging. The majority of the participants exhibited significant improvements in skin quality on the test side compared to the control side, as supported by investigator‐assessed and self‐evaluated GAIS scores. Regarding safety, no adverse events related to the RhCol‐III solution injection were reported. Only one participant experienced mild adverse events (acneiform rash and skin irritation) related to the serum. Symptoms resolved after discontinuing the serum and receiving concomitant medication. These findings indicate that the RhCol‐III solution injection, used either in combination with collagen‐III multi‐peptide serum or as monotherapy, demonstrates both effectiveness and a good safety profile.

In the field of aesthetics, collagen‐based products are extensively utilized to improve skin health, reduce wrinkles, and enhance skin elasticity [1]. Common administration approaches include oral intake, topical application, and intradermal injection [1, 6]. Oral products are absorbed through the digestive system and enter the bloodstream to reach skin cells, promoting endogenous collagen synthesis. Several studies have demonstrated that oral collagen supplements can reduce wrinkles and fine lines while increasing skin elasticity and firmness [17, 18, 19]. However, oral collagen must be digested into smaller amino acids or peptides before being absorbed through the intestinal wall into the bloodstream [19]. This process results in a slower onset of effects and variable absorption efficiency among individuals.

Topical application primarily involves skincare products such as masks and serums, which often contain collagen or collagen peptides intended to improve skin texture and reduce signs of aging. Besides these products, there have also been attempts to directly apply collagen solutions onto the skin. A study utilized fractional ablative CO_2_ laser to vertically ablate micro‐columns through both the epidermis and dermis, facilitating the absorption of topically applied recombinant human collagen, and the results showed significant improvements in various periorbital skin aging indices 3 months post‐treatment [20]. Unfortunately, this study did not include a separate laser‐only control group, introducing potential confounding factors in evaluating the efficacy of topical collagen application. It should be noted, however, that the role of collagen in skincare products is not merely “direct replenishment” but rather leveraging its biochemical properties, such as binding water molecules through its abundant glycine, hydroxyproline, and hydroxylysine residues, thus providing excellent hydration and stimulating fibroblast synthesis of new collagen for anti‐aging benefits [1, 8, 11] Moreover, functional skincare products often incorporate low molecular weight peptides and penetration enhancers to aid in the absorption of active ingredients [1]. Draelos et al. found that peptide‐containing skincare products significantly reduced facial wrinkles at rest and improved wrinkles during maximum smiling in 29 female participants after 14 weeks of follow‐up [21]. In the present study, we applied a collagen‐III multi‐peptide serum on the tested side following the injection. Compared to the injection‐only control side, the test side exhibited significant improvements in skin surface evenness and skin quality 12 weeks post‐treatment. Our findings, supported by a larger sample size and incorporating both objective measurements and subjective assessments, demonstrate that topical collagen peptides have significant effects in improving the appearance of facial skin with mild to moderate photoaging.

Injectable collagen represents a novel breakthrough in aesthetic medicine. Recombinant humanized type III collagen protein solution, upon injection, forms a collagen fiber network that supports cells and tissues, promotes autologous fibroblast synthesis of new collagen, leading to tissue repair, regeneration, and physical filling, thereby firming the skin, repairing wrinkles, and delaying photoaging [14, 15, 17, 22]. Direct collagen supplementation through injection presents advantages over traditional bio‐stimulatory components. Unlike fillers containing hyaluronic acid fragments and amino acids, which rely on the activation degree of fibroblasts for promoting type III collagen synthesis, direct collagen supplementation provides structural support independent of fibroblast activity [23, 24]. By rapidly replenishing lost collagen in the dermis, this method bypasses the uncertainties associated with endogenous synthesis regulation [22]. Moreover, as a primary component of the extracellular matrix, collagen exhibits immediate mechanical improvements in skin elasticity and density, which is particularly crucial in counteracting collagen degradation caused by photoaging [1]. The findings of this study also indicate that RhCol‐III solution injection significantly enhances skin firmness in participants. A prior study conducted by Yang et al. demonstrated that a new dermal injectable collagen exhibited notable efficacy for correcting nasolabial fold wrinkles [22]. Similarly, two other case series by Duan et al. and Tao et al. demonstrated significant improvements in self‐assessed GAIS scores following intradermal injections of RhCol‐III solution, aligning with our findings. In our study, investigator‐assessed GAIS scores indicated a peak efficacy at Week 8, followed by a decline consistent with the transient clinical duration of the collagen product. In contrast, participant‐reported self‐assessments remained relatively stable throughout the follow‐up period, ranging between 53% and 60%. This discrepancy highlights the difference in sensitivity between trained clinical grading and subjective patient perception. Patients may perceive an immediate “improvement” that plateaus, whereas investigators are able to discern subtler gradations of clinical, aesthetic improvements. The two case series, along with our clinical study, highlight RhCol‐III solution's broad‐spectrum effectiveness in enhancing skin quality. Notably, Duan et al. elucidated a plausible biological mechanism for RhCol‐III's therapeutic effects in skin repair: it significantly reduced pro‐inflammatory cytokines TNF‐α and IL‐6 secretion in vitro, suggesting anti‐inflammatory potential. This aligns with chronic inflammation's established role in photoaging [14]. However, despite these promising findings, research on injectable collagen as an anti‐aging treatment remains less extensive compared to oral alternatives. Our study investigated the effectiveness and safety of a RhCol‐III solution injection. By providing additional data, our work aims to enhance the understanding of this delivery method's clinical application within collagen‐based products.

The integrated skincare approach highlights the importance of a combined treatment strategy. While collagen injections can improve skin structure, their combination with topical collagen‐III multi‐peptide serum achieves synergistic effects. Mechanistically, collagen injections provide structural support, whereas the serum, containing small molecule collagen peptides, can continuously stimulate fibroblast activity through epidermal penetration. This “inside‐out and outside‐in” model achieves promising outcomes, as demonstrated by the present study [25]. Additionally, maintaining skin barrier function through daily care aligns with preventive anti‐photoaging medical principles [11]. Our study demonstrated that the side treated with RhCol‐III solution injection followed by the application of a topical collagen‐III multi‐peptide serum for 12 weeks showed greater participant satisfaction, improved surface evenness, and enhanced skin quality compared to the control side, supporting the integrated skincare strategy.

A limitation of this study is the reliance on investigator visual evaluation using the Skin Aging Atlas. While this is a validated clinical tool, it remains inherently subjective. Future research should incorporate objective instrumental analysis methods to provide quantitative data on wrinkle depth and skin roughness, such as 3D fringe projection profilometry or multispectral 3D analysis.

The present work lays the groundwork for subsequent studies to investigate critical aspects of RhCol‐III interventions. First, in real‐world clinical practice, RhCol‐III injections are often combined with other energy‐based devices or injectable products. Thus, studies on such integrated skincare regimens are warranted to explore their synergistic benefits. Second, subgroup analyses (e.g., by Glogau photodamage classification or age) can be conducted to assess heterogeneous treatment responses. Finally, alternative designs (e.g., stratified randomization) could complement this study and provide more comprehensive insights into the efficacy of the integrated skincare approach.

## 5 Conclusion

This clinical trial provides evidence on the effectiveness and safety of RhCol‐III solution injection in combination with collagen‐III multi‐peptide serum in improving photoaging of the forehead, periorbital and cheek areas among Chinese women. The findings also confirm that while injection monotherapy achieves significant anti‐photoaging outcomes, the integrated skincare strategy has an added‐on benefit, offering valuable insights into anti‐aging treatments and contributing to the body of knowledge on aesthetic dermatology for this population.

## Author Contributions

Xiaolei Qin designed and planned the study; Jinlong Zhai, Lingyan Liu, Hong Shu, Lin Zhu, Qi Shi, Yining Luo conducted data acquisition; Jinlong Zhai, Lingyan Liu, Hong Shu, Lin Zhu, Qi Shi, Yining Luo performed data analysis and presentation, Xiaolei Qin, Jinlong Zhai, Lingyan Liu, Hong Shu, Lin Zhu, Qi Shi, Yining Luo wrote the initial version of the manuscript, and all authors reviewed the manuscript, Xiaolei Qin prepared the final version of the manuscript.

## Funding

Study design, overall study protocol, clinical intervention and outcome evaluation conducted by licensed physicians. Funding for this clinical trial was provided by L'Oreal Dermatological Beauty China, Skinceuticals.

## Disclosure

The authors have nothing to report.

## Ethics Statement

The authors have nothing to report.

## Consent

Informed consent was obtained from all individual participants included in the study.

## Conflicts of Interest

The authors declare no conflicts of interest.

## Data Availability

The data that support the findings of this study are available on request from the corresponding author. The data are not publicly available due to privacy or ethical restrictions.
